# Developing a disease-specific annotation protocol for *VHL* gene curation using Hypothes.is

**DOI:** 10.1093/database/baac109

**Published:** 2023-01-06

**Authors:** Dena Salehipour, Kirsten M Farncombe, Veronica Andric, Safa Ansar, Sean Delong, Eric Li, Samantha Macpherson, Sarah Ridd, Deborah I Ritter, Courtney Thaxton, Raymond H Kim

**Affiliations:** Department of Medicine, Division of Medical Oncology, University Health Network, 620 University Ave, Toronto, ON M5G 2C1, Canada; Toronto General Hospital Research Institute, University Health Network, 200 Elizabeth St, Toronto, ON M5G 2C4, Canada; Department of Medicine, Division of Medical Oncology, University Health Network, 620 University Ave, Toronto, ON M5G 2C1, Canada; Department of Medicine, Division of Medical Oncology, University Health Network, 620 University Ave, Toronto, ON M5G 2C1, Canada; Department of Medicine, Division of Medical Oncology, University Health Network, 620 University Ave, Toronto, ON M5G 2C1, Canada; Department of Medicine, Division of Medical Oncology, University Health Network, 620 University Ave, Toronto, ON M5G 2C1, Canada; Department of Medicine, Division of Medical Oncology, University Health Network, 620 University Ave, Toronto, ON M5G 2C1, Canada; Department of Medicine, Division of Medical Oncology, University Health Network, 620 University Ave, Toronto, ON M5G 2C1, Canada; Department of Pediatrics, Baylor College of Medicine and Texas Children’s Hospital, 1102 Bates Ave, Houston, TX 77030, USA; Department of Genetics, University of North Carolina, 120 Mason Farm Rd, Chapel Hill, Chapel Hill, NC 27514, USA; Division of Medical Oncology and Hematology, Princess Margaret Cancer Centre, University Health Network, Sinai Health System, 620 University Ave, Toronto, ON M5G 2C1, Canada; Division of Clinical and Metabolic Genetics, The Hospital for Sick Children, 555 University Ave, Toronto, ON M5G 1X8, Canada; Ontario Institute for Cancer Research, 661 University Ave Suite 510, Toronto, ON M5G 0A3, Canada; Department of Medicine, University of Toronto, 1 King’s College Cir, Toronto, ON M5S 1A8, Canada

## Abstract

Von Hippel–Lindau (VHL) disease is a rare, autosomal dominant disorder that predisposes individuals to developing tumors in many organs. There is significant phenotypic variability and genetic variants encountered within this syndrome, posing a considerable challenge to patient care. The lack of *VHL* variant data sharing paired with the absence of aggregated genotype–phenotype information results in an arduous process, when characterizing genetic variants and predicting patient prognosis. To address these gaps in knowledge, the Clinical Genome Resource (ClinGen) VHL Variant Curation Expert Panel (VCEP) has been resolving a list of variants of uncertain significance within the *VHL* gene. Through community curation, we crowdsourced the laborious task of variant annotation by modifying the ClinGen Community Curation (C3)-developed Baseline Annotation protocol and annotating all published *VHL* cases with the reported genotype–phenotype information in Hypothes.is, an open-access web annotation tool. This process, incorporated into the ClinGen VCEP’s workflow, will aid in their curation efforts. To facilitate the curation at all levels of genetics expertise, our team developed a 4-day biocuration training protocol and resource guide. To date, 91.3% of annotations have been completed by undergraduate and high-school students without formal academic genetics specialization. Here, we present our VHL-specific annotation protocol utilizing Hypothes.is, which offers a standardized method to present case-resolution data, and our biocuration training protocol, which can be adapted for other rare disease platforms. By facilitating training for community curation of VHL disease, we increased student engagement with clinical genetics while enhancing knowledge translation in the field of hereditary cancer.

**Database URL**: https://hypothes.is/groups/dKymJJpZ/vhl-hypothesis-annotation

## Introduction

Von Hippel–Lindau (VHL) disease is a rare, inherited autosomal dominant disorder that predisposes individuals to the development of early-onset tumors in multiple organ systems (1). It has an estimated prevalence of 1 in 31 000–53 000 individuals worldwide and is caused by a germline mutation in the *VHL* tumor suppressor gene (1, 2). Individuals with VHL disease develop multi-organ tumors, including retinal and central nervous system hemangioblastomas, clear cell renal cell carcinoma, pheochromocytomas/paragangliomas (PPGL), renal cysts, pancreatic neuroendocrine tumors, pancreatic cysts, endolymphatic sac tumors and epididymal and broad ligament cysts (1, 3). Disease penetrance is dependent on both the patient’s age and tumor type, and there is significant phenotypic variability both within and between families (1, 3). The onset of VHL disease most commonly occurs within the second or third decade of an individual’s life (mean age of onset 26.3 years of age), with 97% of patients experiencing symptoms by age 60 (3, 4).

After a single member of a family has been identified with a pathogenic *VHL* mutation, cascade genetic testing is initiated to identify additional family members, including asymptomatic carriers (5, 6). The recommendation of life-long clinical surveillance for patients with VHL disease has increased the median life expectancy of patients from 41–49 to 52.5 years of age (6, 7). With increased knowledge regarding the presentation and severity associated with specific *VHL* variants, precision medicine opportunities may exist to tailor guidelines to each individual to improve screening and decrease patient burden (8, 9).

The relatively new integration of genotyping into the clinical setting has improved genetic testing for patients with VHL disease (6, 8). While a large number of disease-causing variants are reported in published literature and databases, information is scattered among various platforms (10, 11). Additionally, variants of uncertain significance (VUS) are commonly encountered, which pose a significant challenge for clinicians when determining the optimal treatment and surveillance strategies, as well as being a source of stress for patients (9, 12). Variant classification is a time-consuming process, hampered for VHL due to the number of variants and a lack of *VHL* variant and clinical phenotype data sharing (13, 14).

Currently, several tools exist for the interpretation and summarization of genetic variants in VHL disease. Clinical Interpretations of Variants in Cancer (CIViC) is an open-access knowledge base supporting global cancer variant interpretation by facilitating community consensus (10). All variants published in the literature and uploaded to CIViC are associated with an evidence statement, which provides a summary of variant-specific information, including associated phenotypes, family history, sampling, genetic testing methodology and functional evidence (15, 16). The Clinical Genome Resource (ClinGen) is a centralized genomic knowledge base that assesses the clinical validity, utility and pathogenicity of a variant through evidence-based consensus from its Variant Curation Expert Panels (VCEP) (17, 18). As a result, the information provided by ClinGen is more variant-focused and oftentimes there is minimal phenotypic information provided by the original sources (17–19). The ClinVar National Centre for Biotechnology Information platform combines interpretation evidence from clinical testing and research laboratories, ClinGen VCEPs and genetic service providers into an archive of genetic variants with potential clinical significance (11, 20). Most of ClinVar’s submissions are variant-focused, and often there is a lack of deep phenotyping provided, which prevents users from developing a comprehensive understanding of the features identified in the individuals evaluated (11, 20). This is partly because many entries are uploaded by laboratories that report a limited amount of patient phenotypic information (11, 20). Finally, the Genome Aggregation Database presents population-level information, such as population-specific allele frequency, the age distribution of variants, variant co-occurrence and ClinVar-reported clinical significance (21, 22). Overall, there is a lack of readily available databases with phenotypic information on multiple patients.

While these tools provide useful variant-specific information for VHL disease, there is currently no central hub housing information from these diverse, unstructured resources. The establishment of a central resource for gathering *VHL* case- and variant-resolution information would decrease the time and burden the VCEP must undertake to resolve VUS and provide final clinical validity classifications (23). To address this, we collaborated with the ClinGen Community Curation (C3) Working Group, specifically the Baseline Annotation effort, to develop a specialized version of their Hypothes.is annotation protocol. The aim was to crowdsource time-consuming elements of the curation process, including the literature search and locating detailed phenotypic information and functional evidence (23–26). Hypothes.is is an open-source annotation tool that allows users to create and view annotations made by others over any published source—analogous to a sticky note—that provides additional information or personal insight (27, 28). An annotation is a string of text that denotes important information extracted from a paper and is associated with a relevant and specific section of the text. Hypothes.is users can create, share, search and reply to annotations that are public or those that are private to themselves and their groups (27–29). Each type of annotation is accompanied by user-specified tags that contribute to the searchability of all pertinent information annotated on Hypothes.is (27). The advantage of Hypothes.is over other existing databases is that individuals/groups can create a standardized data collection that allows a searchable format that is directly associated with the originating source; in our case, it supports the creation of a specific repository of *VHL* annotations. Our aim is to annotate all published germline *VHL* variants through the Hypothes.is interface and to integrate this data with the *VHL* VCEP’s workflow to enhance their curation efforts. Here, we demonstrate how the C3 Baseline Annotation protocol (26) can be adapted for specific diseases and present a biocuration training protocol that can be used to train individuals with a wide range of expertise to successfully annotate genetics literature.

## Methods

The annotated papers were selected through a literature search (30) on Ovid MEDLINE and EMBASE databases using the terms ‘(Von Hippel–Lindau) AND (genetics OR gene mutations)’ and ‘(Von Hippel–Lindau) and (databases)’. These terms were searched for in the title, abstract and index terms of manuscripts published up to October 2019. These searches were compared against VHLdb and the Universal Mutation Database-VHL databases to confirm the search’s effectiveness. The inclusion criteria were (i) reporting germline VHL variants and (ii) patients having VHL-associated phenotypes or fulfilling the criteria for VHL disease.

The C3 Baseline Annotation protocol (26) was customized to include VHL-specific text annotation subcategories and VHL-specific tags while retaining the standard data structures present in the protocol originally. In brief, the C3 Baseline Annotation protocol (26) included two main annotation types: (i) an ‘article information’ annotation that outlined information such as the PMID and the gene name and (ii) a ‘case-individual annotation’ that provided case-specific information, including the variant, associated phenotypes and family history.

**Figure 1.**
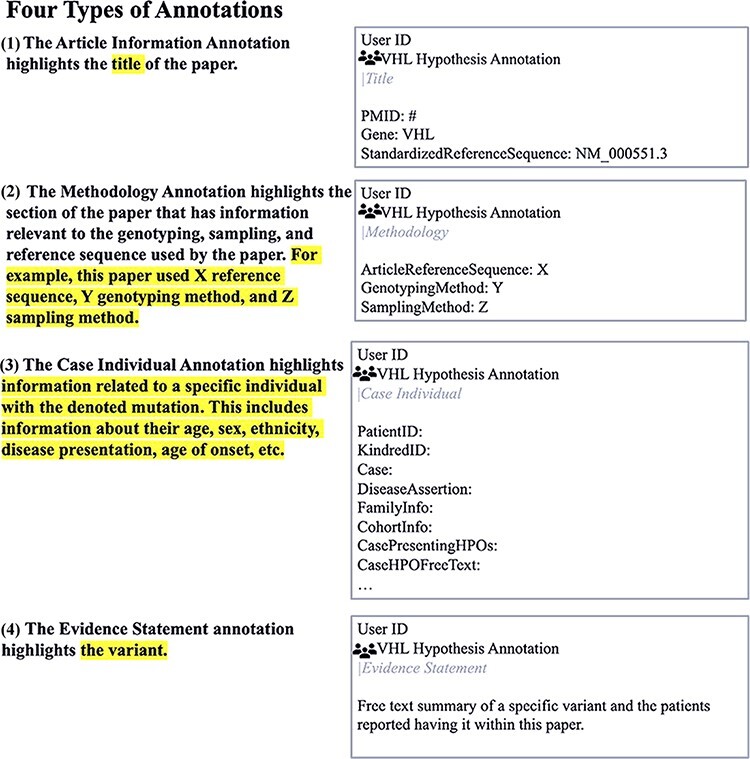
Four types of annotations and their associated text in a sample paper using the Hypothes.is platform.

**Figure 2.**
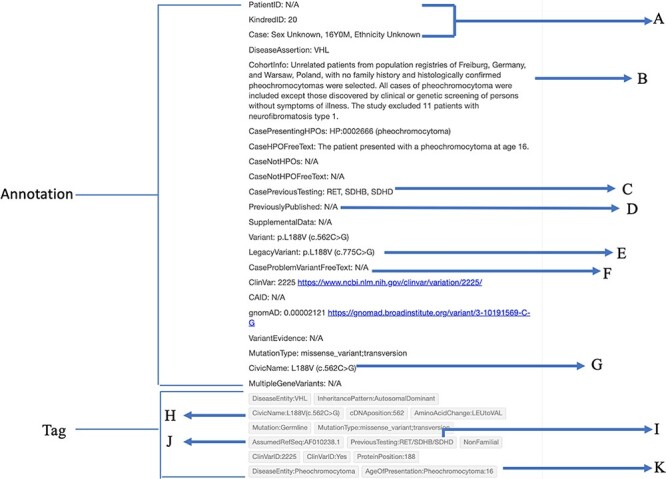
Case-individual type annotation on the Hypothes.is platform. (A) Patient identification information is indicated by patient ID, kindred (family) ID and case information. (B) Description of the cohort. (C) Previous genes that were tested. (D) Previously published. (E) The non-standardized version of the variant, known as the Legacy variant. (F) Problems encountered with the variant. (G) The CIViC name associated with the variant. (H) Tag for the CIViC name. (I) Tag for previous genes that were tested. (J) Tag for the reference sequence used by the authors. In this case, the Latif sequence was assumed. (K) Tag for the age of presentation of a specific phenotype. In this case, a pheochromocytoma presenting at age 16 was tagged.

VHL-specific additions to the ClinGen protocol, also outlined in the ‘Supplementary Material and Methods (VHL Variant Annotation Protocol)’, included the following:

- Four annotation types (Figure 1):
  - Article information: noted identifying information for the article
  - Methodology: outlined the genotyping and sampling methods of the paper
  - Case-Individual OR Group/Cohort-specific: outlined critical information about the variant and affected patients
  - Evidence statement: provided a summary of the paper in relation to a specific variant (adapted from CIViC)
- ‘Kindred ID’, ‘Patient ID’ and ‘Case’ annotations (Figure 2A) were created for identification purposes and easy case counting within a paper.
- ‘CohortInfo’ annotation (Figure 2B) was developed to note specific characteristics shared among the patients in a study’s cohort.
- ‘PreviousGeneticTesting’ annotation (Figure 2C) and ‘PreviousGeneticTesting’ tag (Figure 2I) for any previous testing involving *MAX, NF1, RET, TMEM127, SDHA, SDHB, SDHC, SDHD* and/or *SDHAF* genes. These were included to address that certain phenotypes commonly present in VHL disease (e.g. PPGL) overlap with other genetic syndromes (associated with the above variant list) (31). The inclusion of negative genetic test results increased confidence that PPGL is specifically associated with a *VHL* pathogenic variant, increasing the pathogenicity confidence by the VHL VCEP (31, 32).
- ‘PreviouslyPublished’ annotation (Figure 2D) was created to reduce reports of the same patient in the literature as multiple studies often report on the same cohorts. This was particularly important as the number of mutation carriers is an important criterion for the VHL VCEP’s variant classification efforts (32).
- ‘ArticleReferenceSequence’ and ‘LegacyVariant’ annotations (Figure 2E) and ‘RefSeq’ tag (Figure 2J) were developed to recognize *VHL* variants mapped to older *VHL* transcripts [e.g. Latif *et al.*, 1993 (AF010238.1)] that required standardization to the current genetic reference sequence (NM_000551.3). Having these specialized annotations and tags eased the interpretation of unstandardized variants and allowed easy reference to the published literature.
- ‘CaseProblemVariant’ annotation (Figure 2F) and ‘ProblemVariant:Unresolved’ tag addressed variants with an error in the codon and/or protein change reported in the literature, where our efforts to address these discrepancies were unsuccessful. This expanded on the C3 Baseline Protocols tag ‘UnregisteredVariant.’
- ‘CivicName’ annotation (Figure 2G) and tag (Figure 2H) linked an annotation to a particular CIViC variant entry and cross-linked Hypothes.is annotations to other VHL databases.
- ‘AgeofPresentation’ tag (Figure 2K) noted the age of presentation of a particular phenotype. Since VHL disease has a variable age of presentation, linking genotype–phenotype information with specific ages was critical to improving VHL patient surveillance guidelines (13, 31).

**Figure 3.**
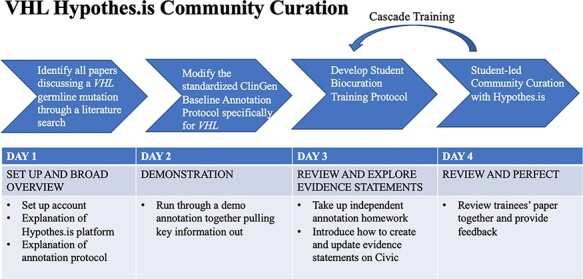
VHL-specific protocol development and training protocol. Summary of a 4-day lesson plan. Refer to Supplementary Material and Methods for the full training protocol, which details the specific steps and homework tasks that were assigned, and the resource guide, which includes links and diagrams that are useful during the annotation process.

VHL annotations with Hypothes.is were facilitated by student annotators at various educational stages. A student training protocol [‘Supplementary Material and Methods (Hypothes.is VHL Group Annotation Training and Toolkit)’], composed of a 4-day lesson plan and a resource toolkit, was developed to provide students of any educational background with a comprehensive understanding of VHL-specific annotation guidelines. This lesson plan took into consideration visual, auditory and tactile learning styles (33) and implemented the following teaching strategies: visual demonstration through an annotation walkthrough, verbal explanations by reading the protocol out loud together and providing a summary, and tactile assessments by assigning students their own articles to annotate and providing intensive feedback. The resource toolkit is composed of an easily accessible list of websites and diagrams that are habitually used during the annotation process (15, 18, 20, 22, 34, 35). Cascade training was implemented for the VHL-specific annotation protocol, with each new set of annotators trained by the previous group of biocurators (Figure 3).

## Results/Discussion

VHL-specific changes to the ClinGen Baseline Annotation protocol were implemented to assist with variant interpretation and curation. By adding these VHL-specific annotations and tags, relevant information was extracted and presented in a standardized, searchable and easily accessible manner that increased the efficiency of variant classification by the VHL VCEP. In comparison with other genetic databases, Hypothes.is provided a more granular, case-specific resolution and served as a hub for connecting a variety of databases. Hypothes.is can be used as an alternative to existing genetic databases or used to complement their data, potentially filling in gaps.

The variability of variant availability across different platforms was apparent, as 50% (231/462) of the annotated variants had a corresponding ClinVar ID, 43% (199/462) only had an associated Canonical Allele Identifier and 6% (28/462) could only be found on CIViC. This is problematic as these existing databases do not encompass all reported variants. Eighty-four cases of unregistered variants (where the mutation denoted by the author was too vague) were encountered. Out of the 430 papers that were extracted, 86% (371/430) have been annotated thus far. Based on previous work by our team (30), we estimated 566 unique variants within these 430 papers; however, we expect this number will fluctuate by the end of the project. Within these 371 papers, there were 458 unique variants. About 30% (111/371) included pedigrees, 15% (57/371) included experimental assays and 43% (159/371) contained previously published cohorts; these three pieces of information are all considered valuable evaluation criteria in the variant curation process. Additionally, 36% (134/371 papers) utilized a non-standardized reference sequence that required standardization.

The VHL Annotation team worked closely with the VHL VCEP to identify VUS within the curated literature that had overwhelming evidence and flagged these as high priorities for annotations. The Hypothes.is tool has been integrated into the VHL VCEP’s workflow, and VCEP members have received training on how to best use the tool to enhance their curation efforts. Hyperlinks to the VHL Hypothes.is Annotations are also available in the ClinGen Variant Curation Interface (36), through the Hypothes.is application programming interface, and can be accessed by any registered user. Through this work, the VHL Annotation team aimed to increase variant classification efforts by crowdsourcing time-consuming components of the work, with the ultimate goal of supporting the creation of clinically useful expert-level curations for all *VHL* variants reported in the literature.

Since the inception of the VHL Annotation team in September 2019, the group has performed 8629 annotations (Table 1), where 59.8% of annotations have been completed by undergraduate Bachelor of Science students, 13.1% by Masters’ students in Medical Genomics, 24.5% by an undergraduate Bachelor of Arts student and 2.6% by a high-school student. The knowledge translation system was dependent on an easily understandable, comprehensive protocol and an open-communication system where students deliberated issues weekly in a collaborative environment and ensured the group acted cohesively in their approach to challenging annotations. Additionally, this facilitated concurrent quality assessment of work. The training protocol was a key element in ensuring consistent annotation practices across the team. Quality control was confirmed by pairing a senior annotator with a more novice one, and through the feedback provided, we saw a reduction in the number of mistakes and an increase in consistency.

**Table 1.** Hypothes.is annotation and tag breakdown (November 2022)

Users	Number of annotations	Top 20 tags overall
denasalehipour ericli3 ridds Safa_Ansar netapipko samantha_macpherson veronicaandric Total	1102 222 4060 558 439 131 2114 8629	Mutation:Germline InheritancePattern:AutosomalDominant DiseaseEntity:VHL ClinVarID:Yes Familial EvidenceStatement AssumedRefSeq:NM_000551 ClinVarID:N/A MutationType:missense_variant; transition DiseaseEntity:hemangioblastoma NoFamilyInfo DiseaseEntity:renalcellcarcinoma DiseaseEntity:pheochromocytoma DiseaseEntity:retinalcapillaryhemangioma MutationType:missense_variant; transversion SupplementalData FamilyPedigree UnregisteredVariant AssumedRefSeq:AF010238 RefSeq:AF010238

The experience of biocuration was a fantastic learning experience for the students on the Hypothes.is team. Every student chose to remain longer on the project than their original work term. The students described developing an understanding of different genetic databases and genotype logistics and enhancing their level of confidence reading and summarizing scientific literature as reasons that prompted their continuation with the team (personal communication). Biocuration from an early career stage is highly beneficial, as students become proficient in understanding and extracting information from scientific literature, analyzing data and summarizing key information. Community curation is a powerful tool that can be used to increase the amount of publicly available genetic data to accelerate the evaluation of VUS and improve variant curation efforts.

## Conclusion

By adapting existing resources for open-source annotation of genetic information through the use of Hypothes.is, a freely available annotation tool, we created a standardized repository of case-resolution genetic data to aid in variant classification. Introducing annotation as an element of biocuration was able to address gaps encountered with other variant-resolution platforms, allowing for greater knowledge transmission and increased engagement of the younger generation as biocurators. As evidenced by the diverse educational background of members of the VHL Annotation team, biocuration is universally accessible. The 4-day training protocol and resource guide developed by our team can serve as a reference to be adapted for other individuals interested in engaging new trainees in the process of biocuration, including those with an interest in contributing to clinical genetics and the genetics of rare diseases. Integrating annotation into the VHL VCEP’s workflow positively contributed to the VCEP’s goals for variant curation. Our work showed that annotation may be beneficial for various groups undergoing data assessment for variant interpretation, both within ClinGen and by independent curation efforts.

## Supplementary Material

baac109_Supp

## Data Availability

The data discussed in this study has been uploaded on to the Hypothes.is web interface (https://hypothes.is/groups/dKymJJpZ/vhl-hypothesis-annotation).
