# Supplementary material for: Developing a disease-specific annotation protocol for VHL gene curation using Hypothes.is
Source: Database (Oxford). 2023 Jan 6;2023:baac109. doi: 10.1093/database/baac109 (PMC9825735; doi:10.1093/database/baac109)
Supplement: baac109_Supp [file baac109_supp.zip › suppl_data/Supplementary Material and Methods.docx]

**Supplementary Material and Methods**

**Section 1: VHL Variant Annotation Protocol**

This protocol is to outline the evidence, tags, and procedures to follow for the annotation of variants associated with the gene [*VHL*](https://www.omim.org/entry/608537?search=VHL&highlight=vhl) (OMIM info in hyperlink).

**VHL standardized reference sequence (FASTA):**

**NM000551.3:** <https://www.ncbi.nlm.nih.gov/nucleotide/NM_000551.3>

**CCDS2597.1:** <https://www.ncbi.nlm.nih.gov/CCDS/CcdsBrowse.cgi?REQUEST=CCDS&DATA=CCDS2597.1>

- Coding sequence ONLY with intron-exon demarcation and protein translation

**Hypothesis VHL Variant curation group link:**<https://hypothes.is/groups/dKymJJpZ/vhl-hypothesis-annotation>

**Hypothesis S.O.P. (Standardized Operating Procedure, ClinGen, July 2021):**

<https://clinicalgenome.org/site/assets/files/3875/hypothesis_gene_annotation_sop_version_3.pdf>

**Steps for annotating:**

**Step 1:**  Ensure that the article selected for annotation fulfills the following criteria.

- Assess if there are assertions of case(s)/individual(s)/proband(s) identified with a variant in *VHL*
  - Presents a novel patient study (e.g. no literature reviews)
  - Confirmed germline VHL variant
  - VHL mutation specifications (e.g. c.74C>T or equivalent)
  - Phenotype data pertaining to a case (patient) that has asserted variant(s) in VHL

**Step 2:** Perform annotations.

The table below represents two separate annotations: (1) Article information annotation, in which the title of the article is highlighted; (2) Methodology annotation, in which the methods of the paper are outlined; (3) Case-Individual **OR** (3) Group/Cohort specific annotation, in which all the pertinent evidence on a particular case are outlined.

**When searching on Hypothes.is:**

- Tags may be used in combination with one another
  - **For example**: “c.194C>G” and “Familial” will provide a narrower search than “c.194C>G”
- Both tags and text annotations will appear in search results if the appropriate term is pulled

**Supplementary Table 1. Case report example.**

| **Annotation Category (tags to include)** | **Text to highlight** | **Information to outline within the annotation report** |
| --- | --- | --- |
| Article Information | Title of article | **PMID:** add PMID or article  **Gene:**  add [HGNC](https://www.genenames.org/) name  **StandardizedReferenceSequence:** NM_000551.3  (Variants documented on annotation report should have standardized nomenclature with c. and p. be converted to the most current reference sequence, e.g. For VHL, the most current reference sequence is NM_000551.3) |
| Methodology | Text specifying how mutation information was determined for the study (what sample was collected, how was the mutation detected, or was the information collected form the patient’s chart and if that was the case, whether the mutation was confirmed) | **ArticleReferenceSequence:** Annotate with the reference sequence used by the authors to determine the variant, this could be one of the older versions of the reference sequence for the gene ,(e.g. NM_000551.2, NM_000551.1, **AF010238.1 (Latif et al. Legacy sequence)**, L15409.1)  ***Note:*** if reference sequence was not specified in the article, annotate with “Not Specified” along with assumption (e.g. Not specified – Assumption: variants assumed to have been documented by the author under reference sequence NM_000551.3)  If you encounter a problem variant where for instance the c. position must be inferred from the amino acid position you can explain this: “Not specified- Assumption: Nucleotide change is not specified in the paper, only protein coding change is. cDNA variant was inferred based on protein coding change and reported based on VHL reference sequence NM_000551.3.”  **GenotypingMethod:** Annotate with the method used to determine the genotype if known. E.g. Whole exome sequencing (WES). Include any methods used to genotype other genes in the paper.  **SamplingMethod:** annotate with the genome sampling method (e.g. peripheral blood). If no sample is mentioned, put N/A |
| Case Individual    (See Table notes below, and Table 3 for applicable tags to add). | Text asserting the case/individual/proband with genetic variation in the gene of interest. | **PatientID:** Annotate with the individual label used for the patient in the paper.  > If there is no patient ID listed in the paper, put: ‘None’  **KindredID:** Annotate with kindred Case ID used in the paper or a simple integer id where this is missing. Can be arbitrarily assigned if not in paper- this is used to link families. This field is present in the final masterlist under “Kindred Case”. **If the patient is not linked to a family, use an arbitrary integer value (found in the masterlist.)**  **Case:** sex, age, ethnicity if known. If any are unknown, write ‘Sex Unknown’, ‘Age Unknown’, or ‘Ethnicity Unknown’ in order. For example: “Case: F, 45Y0M, Ethnicity Unknown”  *Note: if multiple ages are listed in the paper, just put the last reported age.  **DiseaseAssertion:** Annotate with the HPO TERM disease assertion made by the authors for the proband/case of interest   - **If clinically diagnosed:**  DiseaseAssertion: VHL - **One phenotype mentioned, or tumor resolution study:** DiseaseAssertion: Pheochromocytoma - **Multiple (2+) VHL relevant phenotypes reported, but clinical diagnosis not explicitly stated:** DiseaseAssertion: Assumed VHL - **Paper indicates asymptomatic:**   DiseaseAssertion: Asymptomatic  **FamilyInfo:** **Use free text** to annotate with information on the family including descriptive mention in text, figure or table number, and demographic label, and patient ID’s are included. **Add pedigree figure #.**  **Example: *Case II:3 (the patient’s 21 year old brother) had bilateral pheochromocytoma. Case II:4 the patient’s sister (F) had renal cell carcinoma, bilateral pheochromocytoma, cerebral hemangioblastomas, and pancreatic cysts. Case II: 5 the mother (died 43Y, F) had pancreatic cancer. Uncle had an operation for pheochromocytoma, and second uncle died of a spinal tumor. Pedigree: see Figure 2.***  ***CohortInfo: Use free text** to annotate with information on the cohort the individual patient is part of.  ***ONLY INCLUDE IN THE FOLLOWING SPECIAL CASE: Tumor resolution studies with patient level information. Example annotations: PMID 22156657.**  **CasePresentingHPOs:** Annotate with the [HPOs](http://compbio.charite.de/hpoweb/showterm?id=HP:0000118) presenting specifically in the proband of interest. (See VHL phenotype HPO chart)  Format: *HP:0006748, HP:0009711 (adrenal pheochromocytoma, retinal capillary hemangioma)*  **CaseHPOFreeText:** Annotate with phenotype information that does not have an appropriate HPO number, or requires explanation (i.e. include phenotype descriptions here including **age of presentation** if mentioned in the paper**).**  **CaseNotHPOs:** Annotate with the [HPOs](http://compbio.charite.de/hpoweb/showterm?id=HP:0000118) that were noted **not** to manifest within the proband of interest.   - **“None”** if they looked at all phenotypes and found none of them presented in the patient - **“N/A”** if none mentioned (they did not rule out phenotypes explicitly in the paper)   **CaseNotHPOFreeText:** Annotate with phenotyping information that appears normal or unaltered in the patient, yet does not have an appropriate HPO number, or requires explanation.  **CasePreviousTesting:** If other genes of interest to the disease were tested, list here. If they did a panel of genes, note the panel used and any reference to the gene list (i.e. table that lists genes on panel, or the name of the panel used).  **(only these:)** MAX  NF1  RET  TMEM127  VHL  SDHA/SDHB/SDHC/SDHD/SDHAF2  (or panel name- if they mention it)  **PreviouslyPublished:** Annotate with the PMID(s) if known (usually stated in paper).  **SupplementalData:** Annotate with the identifying table, figure, of file number(s) where the case information was outlined in the supplemental.  Example: “Information obtained from Table S1 and Table S2”    **Variant:** Annotate with the variant after standardized with current reference transcript.  ***Note:*** Record non-specific exon loss variants as:   - Exon 1 loss - Exon 1-2 loss - etc.   ***If*** the paper uses proper HGVS nomenclature for breakpoints, include in brackets after ‘Exon _ loss’.   - Ex. of what you would see: **c. 341-?_462+?del** *(a deletion of exon 2 with unspecified intronic breakpoints)* - Exon 1 is c.1-340 - Exon 2 is c.341-462 - Exon 3 is c.463-642   ***Note:*** If standardization could not be performed under protocol, annotate with “Unreliable Variant Standardization”. This is for **problem variants** *(liftover not working, only legacy available)*  ***Note:*** *For autosomal recessive inheritance or compound heterozygous mutations, you will need to separate variant entries and provide both ClinVar IDs or CA IDs.*  **LegacyVariant:** Annotate with variant as it has been documented in the paper (before lift-over), if different from standardized variant  **CaseProblemVariantFreeText:** Annotate with problem variant status/ method used to solve variant *Put this information in if you can explain how a previous problem variant was resolved  **Annotate the variant with EITHER the ClinVarID, OR the CAID, with the ClinVar ID taking precedence. If there is no ClinVarID, use the existing CAID or create a new CAID** [**(see here)**](https://www.clinicalgenome.org/docs/using-the-clingen-allele-registry/)  **ClinVarID:**  If known, use the ClinVar Variation ID!! (Do not use the allele ID!! Or any other ID! The variation ID is also in the URL.) Link the URL for this page. Make sure that you aren't putting the Clinvar ID for the legacy variant! Ex. 428804, <https://www.ncbi.nlm.nih.gov/clinvar/variation/428804/>  **CAID:** If no associated ClinVar ID, register the variant with the [ClinGen Allele Registry](http://reg.clinicalgenome.org/redmine/projects/registry/genboree_registry/landing) (CA) and copy or create a CA ID. **Include the URL.** **Note:** if there is no CAID we will have to go through the process of registering this.  **gnomAD:** Annotate with the **total population allele frequency** for the variant in question if found in [gnomAD](http://gnomad.broadinstitute.org/), and **add the URL** for the specific variant page.  *Note: this annotation is *static* and we are only linking to gnomAD version 2.1.1. The expert panel must note to double-check the gnomAD **non-cancer** database for more accurate population statistics.  **VariantEvidence:** This is a free text annotation.  Annotate with any information provided in the paper on the variant function, expression levels, activity etc. Label with any applicable figure or table that outlines the evidence. If the paper refers to another study, include the PMID.  **MutationType:** Annotate with information concerning the type of mutation, including missense, deletion, insertion, transversion, etc. See Table 2 “Mutation Type” below for more details.  **CivicName:** Annotate the name that will be used when uploading data to CIViC. See table 2 for specifications .    **MultipleGeneVariants:** Annotate when genetic variation in other genes are noted in the case.  Example: “Germline, RET, c.900G>A (pThr580Val)”  Example: “Somatic, VHL, c.456A>T (p.Val230Ser)” **-> for a second variant in the VHL gene we MUST give it its own annotation as well.** |

**Supplementary Table 2. Group report (2+ patients).** Use “GroupID” in place of “KindredID” and “PatientID” when there are 2+ individual cases grouped in a study and you are unable to distinguish differences among them. For example, tumor resolution studies.

| **Annotation Category (tags to include)** | **Text to highlight** | **Information to outline within the annotation report** |
| --- | --- | --- |
| Article Information | Title of article | **PMID:** add PMID or article  **Gene:**  add [HGNC](https://www.genenames.org/) name  **StandardizedReferenceSequence:** NM_000551.3 |
| Methodology | Text specifying how mutation information was determined for the study | **ArticleReferenceSequence:**  **GenotypingMethod:**  **SamplingMethod:**  ***refer to supplementary table 1 for a more detailed explanation** |
| Group/Cohort  See Table notes below, and Table 3 for applicable tags to add). | Text asserting the group/cohort with genetic variation in the gene of interest. This could include:  >Tumor-resolution study  >Family-resolution study (families grouped and no individual phenotypic info) | **GroupID/KindredID:** ONLY if papers list groups of families  **GroupID:** ex. III-2, or 'None'  > Use only if study lists cohorts by any ID  > If there is no group ID listed in the paper, put: ‘None’  **GroupSize:**  ex. 125  **DiseaseAssertion:** Annotate with the HPO TERM disease assertion made by the authors for the proband/case of interest   - **If clinically diagnosed:**  DiseaseAssertion: VHL - **One phenotype mentioned, or tumor resolution study:** DiseaseAssertion: Pheochromocytoma - **Multiple (2+) VHL relevant phenotypes reported, but clinical diagnosis not explicitly stated:** DiseaseAssertion: Assumed VHL - **If indicated by paper as asymptomatic:**   DiseaseAssertion: Asymptomatic  **GroupNumberMales:** #  **GroupNumberFemales:** #  **GroupEthnicity:** ex. Caucasian  **GroupAgeRange:** ex. 18-45  Include if this information is present. Otherwise, all four of the above fields are **N/A.**  **GroupInfo: (Free text)**  **> Any other information/ details**  > If possible, briefly describe how people are grouped.  > Ex. For tumor-resolution studies (studies that group patients in a cohort by specific tumor presentation)  > Ex. For papers that list X number of patients with a certain mutation (groups patients by mutation)  **GroupPresentingHPOs:** Annotate with the [HPOs](http://compbio.charite.de/hpoweb/showterm?id=HP:0000118) presenting specifically in the proband of interest. (See VHL phenotype HPO chart)  Format: *HP:0006748, HP:0009711 (adrenal pheochromocytoma, retinal capillary hemangioma)*  **GroupHPOFreeText:** Annotate with phenotype information that does not have an appropriate HPO number, or requires explanation (i.e. include phenotype descriptions here including age of presentation if mentioned in the paper).  **GroupNotHPOs:** Annotate with the [HPOs](http://compbio.charite.de/hpoweb/showterm?id=HP:0000118) that were noted **not** to manifest within the proband of interest.   - **“None”** if they looked at all phenotypes and found none of them presented in the patient - **“N/A”** if none mentioned (they did not rule out phenotypes explicitly in the paper)   **GroupNotHPOFreeText:** Annotate with phenotyping information that appears normal or unaltered in the patient, yet does not have an appropriate HPO number, or requires explanation.  **GroupPreviousTesting:** If other genes of interest to the disease were tested, list here. If they did a panel of genes, note the panel used and any reference to the gene list (i.e. table that lists genes on panel, or the name of the panel used).  **(only these:)** MAX  NF1  RET  TMEM127  VHL  SDHA/SDHB/SDHC/SDHD/SDHAF2  (or panel name- if they mention it)  **PreviouslyPublished:** Annotate with the PMID(s) if known (usually stated in paper).  **SupplementalData:** Annotate with the identifying table, figure, of file number(s) where the case information was outlined in the supplemental.  Example: “Information obtained from Table S1 and Table S2”    **Variant:** Annotate with the variant after standardized with current reference transcript.  ***Note:*** If standardization could not be performed under protocol, annotate with “Unreliable Variant Standardization”. This is for **problem variants** *(liftover not working, only legacy available)*  ***Note:*** *For autosomal recessive inheritance or compound heterozygous mutations, you will need to separate variant entries and provide both ClinVar IDs or CA IDs.*  **LegacyVariant:** Annotate with variant as it has been documented in the paper (before lift-over), if different from standardized variant  **CaseProblemVariantFreeText:** Annotate with problem variant status/ method used to solve variant *Put this information in if you can explain how a previous problem variant was resolved  **Annotate the variant with EITHER the ClinVarID, OR the CAID, with the ClinVar ID taking precedence. If there is no ClinVarID, use the existing CAID or create a new CAID** [**(see here)**](https://www.clinicalgenome.org/docs/using-the-clingen-allele-registry/)  **ClinVarID:**  If known, use the ClinVar Variation ID!! (Do not use the allele ID!! Or any other ID! The variation ID is also in the URL.) Link the URL for this page. Make sure that you aren't putting the Clinvar ID for the legacy variant! Ex. 428804, <https://www.ncbi.nlm.nih.gov/clinvar/variation/428804/>  **CAID:** If no associated ClinVar ID, register the variant with the [ClinGen Allele Registry](http://reg.clinicalgenome.org/redmine/projects/registry/genboree_registry/landing) (CA) and copy or create a CA ID. **Include the URL.** **Note:** if there is no CAID we will have to go through the process of registering this.  **gnomAD:** Annotate with the **total population allele frequency** for the variant in question if found in [gnomAD](http://gnomad.broadinstitute.org/), and **add the URL** for the specific variant page.  **VariantEvidence:** This is a free text annotation.  Annotate with any information provided in the paper on the variant function, expression levels, activity etc. Label with any applicable figure or table that outlines the evidence. If the paper refers to another study, include the PMID.  **MutationType:** Annotate with information concerning the type of mutation, including missense, deletion, insertion, transversion, etc. See Table 2 “Mutation Type” below for more details.  **CivicName:** Annotate the name that will be used when uploading data to CIViC. See table 2 for specifications.    **MultipleGeneVariants:** Annotate when genetic variation in other genes are noted in the case.  Example: “Germline, RET, c.900G>A (pThr580Val)”  Example: “Somatic, VHL, c.456A>T (p.Val230Ser)” **-> for a second variant in the VHL gene we MUST give it its own annotation as well.** |

**Supplementary Table 3. Searchable tags to add to case-individual or group reports, where applicable.** Do not leave spaces.

| **Category** | **Examples** | **Tag Choices** |
| --- | --- | --- |
| **Gene** | VHL | Gene:VHL |
| **Inheritance Pattern**  ***Note:** For de novo, tagging is based solely on the assertions made by the author(s). Consider adding a note under the “**FamilyInfo:”** subannotation (Table 1 and 3) to include whether confirmatory testing of parents was performed, including maternity and paternity. | Autosomal Dominant  AutosomalRecessive  De novo*  Ethnicity | InheritancePattern:AutosomalDominant  InheritancePattern:AutosomalRecessive  InheritancePattern:DeNovoAssertion  **Ethnicity:** Add what the paper indicates (use gnomad ethnicity naming standards) |
| **Disease Entity**  If more than one disease entity is asserted for the proband please add all applicable tags using HPO terms. | Von Hippel-Lindau (VHL)  Renal cell Carcinoma, somatic (RCC-s)  Pheochromocytoma (Pheo)  Hemagioblastoma, cerebellar, somatic (Hemangioblastoma-s)  No disease asserted | DiseaseEntity:VHL  DiseaseEntity:renalcellcarcinoma  DiseaseEntity:pheochromocytoma  DiseaseEntity:cerebellarhemangioblastoma  DiseaseEntity:asymptomatic |
| **Age Of Presentation** | AgeOfPresentation:diseaseentity:# | AgeOfPresentation:renalcellcarcinoma:24 |
| **Mutation**  To indicate Germline versus Somatic according to authors assertion. | Germline  Somatic | Mutation:Germline  Mutation:Somatic |
| **Variant Standardization**  Reference Sequence and Assumptions | (1) Reference Sequence specified in article  **OR**  Article and variant standardized and published in review O’Brien 2010  (2) Assumption on the reference sequence used in article  (3) **ProblemVariant:** annotate with this tag ONLY if the variant needs to be corrected in any way in the future (waiting for authors to confirm,) or if the variant standardization was done by the Hypothesis curator | (1) RefSeq:######  RefSeq:O’Brien2010    (2) AssumedRefSeq:######  (3) ProblemVariant:Unresolved  Refer to variant standardization protocol for more information on ‘liftover’, aka. standardizing problem variants for transcript NM_000551.3. |
| **Variant**  Prioritize annotating with a ClinVar ID before a CAiD, however only one should be annotated per variant. | (1) cDNA position of the variant (after standardization), DO NOT tag if standardization cannot be performed  (2) Protein sequence position of the variant (after standardization), DO NOT tag if standardization cannot be performed  (3) Amino acid substitution for missense mutation (e.g.LEUtoVAL)  (4) ClinVar ID- please annotate with the number  (5) ClinGen Allele Registry ID- CAiD  (6) Please also label cases in which you are unable to determine the variant ID  (7) For cases that have multiple *VHL* variants  (8) For cases that have multiple independent gene mutations listed | (1)* cDNApositon: ###  cDNAposition:286*  cDNAposition:N/A  *Do not include for **splice site variants.**  ***Deletions:** tag the first cDNA position deleted. Ex. for c.227_229del, *cDNAposition:227*  ***Insertions:** tag the cDNA position 5’ to the insertion sequence. Ex. c.227insTCT, *cDNAposition:227*  ***Duplications:** tag the mRNA position directly subsequent (3’) to the last position copied in the duplication. Ex. c.227_229dup, *cDNAposition:230*  (2) ProteinPosition: ### ProteinPosition:96  ProteinPosition:N/A  ***Insertion:** tag the first protein position noted  ***Deletion:** tag the first protein position noted  (3) AminoAcidChange:[VA1] [YG2] XXXtoXXX AminoAcidChange:GLNtoTER  AminoAcidChange:N/A  (4) ClinVarID:##### ClinVarID:428804  (AND) ClinVarID:Yes  ClinVarID:N/A  (5) CAID:CA##### CAID:CA351750811  (6) UnregisteredVariant  (7) MultipleVHLVariants  (8) MultipleGeneVariants |
| **Mutation Type**  To indicate the type of mutation (e.g. missense, transversion, deletion, etc) using Sequence Ontology terms. | deletion  Frameshift deletion: minus_1*_*frameshift_variant  OR  minus_2*_*frameshift_variant  Frameshift insertion: plus_1_frameshift_variant  OR  plus_2_frameshift_variant  In frame deletion: inframe_deletion  In frame insertion: inframe_insertion  missense_variant;transition missense_variant;transversion  stop_gained  stop_lost  duplication  splice_site_variant  rearrangement_region  UTR (3’): 3_prime_UTR_variant  UTR (5’): 5_prime_UTR_variant  Synonymous_variant  exon_loss_variant  intron_variant  delins | MutationType:deletion  MutationType:splice_site_variant  MutationType:missense_variant;transition  etc.    E.g. A tag of “MutationType:missense_variant;transition” indicates a missense mutation that is also a transition. For multiple choices separate the choices with a “;” (semicolon).  If more than one tag is relevant (rare)- ex. a stopgain delins- include both. |
| **CIViC Name**  This will indicate the amino acid change (foregoing the initial “p.”) using 1-letter symbols and referencing codon positions, followed by the DNA sequence change in brackets. | P25L(c.74C>T) | CivicName:*amino acid change + HGVS DNA change*    E.g. “CivicName:P25L(c.74C>T)” |
| **Previous Testing**  This specifies other genes of interest that had been tested. (e.g. CCND1 which can be a modifier of the disease VHL) | MAX  NF1  RET  TMEM127  VHL  SDHA/SDHB/SDHC/SDHD/SDHAF2 | ***Note:*** Combinations of the choices may be used and are dependent on the information provided for each case. Always begin tag with “PreviousTesting:”    E.g. A tag of “PreviousTesting:MAX/NF1/TMEM127” indicates that each of these genes were tested for variants. For multiple choices separate the choices with a “/” (forward slash).    **Please only indicate previous testing on genes in the column at the left.** |
| **Family Information** | (1) For cases that outline a family pedigree  (2) For cases asserted by authors to have a family history with no pedigree or descriptive info beyond “family history”  (3) For cases where no family history is confirmed by the paper  4) For cases where the paper doesn’t state anything about family history  (5) For cases where parents are sequenced and confirmed to not have the proband’s mutation | 1. FamilyPedigree   AND/OR  (2) Familial  (3) NonFamilial  (4) NoFamilyInfo  (5) deNovoConfirmed  (6) CompoundHeterozygous  (7) FamilyCohort (Group annotation) |
| **Supplemental Data** | If you find that the proband(s) variant information is listed strictly in the Supplemental data, tag each proband. **Proband Case Report (Table 1) can be made as a Page Note in Hypothes.is on the main text.** | SupplementalData |

**Notes on using certain tags:**

- If multiple *VHL* variants are asserted for the case/individual/proband, tag with ***“MultipleVHLVariants”*** and provide the Variant HGVS nomenclature, ClinVar ID or CAiD, and gnomAD frequency for each variant. Create separate Hypothes.is annotations for each variant, and one Hypothes.is evidence statement for both variants (with two ClinVarIDs or CIViC IDs.)
  - **CIViC: prefers combining entries where patients present with multiple VHL variants.** Ex. CivicName:R200W(c.598C>T)andR161Q (c.482G>A).
    - If CIViC does this, annotate with this CIViC name and update the one CIViC entry for both variants. If CIViC does not do this (ie. has separate entries for each variant) follow that.
- If patient variant information is restricted to the supplement, and there is no reference to the patient in the main text of the article, proceed with generating a case report annotation as a Page Note in the Hypothes.is annotation application (Table 1 above).
  - Add the tag “**SupplementalData**” to the page note for the applicable case report(s).
  - Indicate the supplemental file number, table, or figure in which the information was found in the Case-Report annotation text box.

**Supplementary Table 4. Experimental assay and evidence statement.**

| **Category** | **Text to highlight** | **Information to include** | **Tags** |
| --- | --- | --- | --- |
| Experimental Assay | Relevant experimental evidence | No text required. | “ExperimentalAssay”  “ClinVarID:#####” Ex. ClinVarID:428804 OR  “CAiD:CA#####” Ex. CAiD:CA351750811 |
| Evidence Statement | Variant of interest text | The Evidence Statement is meant to be a textual string of 75-125 words. Information that should be included are as follows:   - Cohort data (i.e. all patients in the study presented some common clinical manifestation) - Patient data (i.e. age, sex, ethnicity, variant, phenotype specifications, age of onset of symptoms, etc.) - Relationship to proband in the case of a family - Method of sampling - Type of sequencing - Original version number used in the paper and the original variant as specified in the paper - Outline pathogenicity of the variant referencing the ACMG criteria from Richards et al., (link above) including supporting evidence from the paper   **Do not include: Conclusions arrived at by the authors**    E.g. Genotype-phenotype correlations of 573 VHL patients from 200 kindreds were analyzed. Higher risk of pheochromocytoma was associated with missense mutations that result in substitution of a surface amino acid (p<=0.004) than for patients with missense mutations that disrupt the structural integrity of the VHL gene product (pVHL) or other loss of function mutations (either germline deletions or truncating mutations). This missense mutation, affecting residue within the pVHL protein core, was found in a VHL patient with cerebellar hemangioblastomas. ACMG codes as follows: Supporting evidence of pathogenicity because Missense variant in a gene that has a low rate of benign missense variation and where missense variants are a common mechanism of disease.  **Multiple VHL Variants:**  If multiple *VHL* variants are asserted for the case/individual/proband, tag with ***“MultipleVHLVariants”*** and provide the Variant HGVS nomenclature, ClinVar ID or CAiD, and gnomAD frequency for each variant. Create separate Hypothes.is annotations for each variant, and one Hypothes.is evidence statement for | EvidenceStatement  ClinVarID:###  OR  CAiD:###  OR  associated with the variant.)  3. Civic Name:  (***ONLY*** if there is neither a ClinVar ID or CAiD)  3. ProblemVariant |

**Section 2: Hypothes.is VHL Group Annotation Training and Toolkit**

|  | **Lesson Plan** | **Homework for Trainees** |
| --- | --- | --- |
| **Day 1** | - Share your group’s Hypothes.is invite link to allow trainees to set up their Hypothes.is accounts - Run through the Hypothes.is protocol (see document in Section 1) step-by-step.   - Learn the difference between a ‘tag’ and an ‘annotation’   - Note down the various databases that variant-specific information is added from.   - Learn what an ‘evidence statement’ is. - Assign a sample paper, one that includes:   - Case-Individual annotation   - Some family information   - Legacy variant (if applicable) | - Re-read the VHL annotation protocol in its *entirety.* - Read sample paper in advance of Day 2 (sample paper will be used for the next lesson’s demo annotation). |
| **Day 2** | - Run through a demo annotation together and search for information in the sample paper together.   - Includes re-performing HPO term, ClinVar, ClinGen, CIVIC, and gnomAD searches | - Each trainee will be assigned one variant or case from the sample paper to annotate as ‘homework’. Instructors will go over the annotations with trainees on day 3. |
| **Day 3** | - Take up independent annotation ‘homework’ - Introduce an overview of how to update evidence statements on CIVIC | - Implement feedback received during the session on ‘homework’ from day 2 - Once trainees are comfortable with the protocol, they can each start annotating their own paper. |
| **Day 4** | - Review trainees’ assigned paper together as a team and provide feedback - Continue to monitor and review trainees’ annotations for the next five days | - Implement feedback received during the session on ‘homework’ from day 3 |

*Note: please bring up any difficult annotations during group meetings and they will be reviewed and decided upon as a team.

**Resource Toolkit**

| Purpose | Website | Link |
| --- | --- | --- |
| To find HPO terms associated with a case’s presentation | Human Phenotype Ontology database | <https://hpo.jax.org/app/> |
| To determine how to denote a mutation | HGVS Website of nomenclature | https://varnomen.hgvs.org/ |
| To check the protein change produced by a coding sequence change | Variant Validator | https://variantvalidator.org/ |
| To find the ClinVar Variation ID | ClinVar Database | <https://www.ncbi.nlm.nih.gov/clinvar/> |
| To find the total population allele frequency | Gnomad Database | <https://gnomad.broadinstitute.org/> |
| To find the CAID (only note when there is no ClinVar Variation ID) | ClinGen Allele Registry | <http://reg.clinicalgenome.org/redmine/projects/registry/genboree_registry/landing> |
| Where you upload or edit the evidence statement | Civic Database | <https://civicdb.org/home> |

Amino Acids Nomenclature Reference

| Amino Acid | 3 Letter Code | 1 Letter Code |
| --- | --- | --- |
| Alanine | Ala | A |
| Aspartic Acid | Asp | D |
| Cysteine | Cys | C |
| Glutamic Acid | Glu | E |
| Phenylalanine | Phe | F |
| Glycine | Gly | G |
| Histidine | His | H |
| Isoleucine | Ile | I |
| Lysine | Lys | K |
| Leucine | Leu | L |
| Methionine | Met | M |
| Asparagine | Asn | N |
| Proline | Pro | P |
| Glutamine | Gln | Q |
| Arginine | Arg | R |
| Serine | Ser | S |
| Threonine | Thr | T |
| Valine | Val | V |
| Tryptophan | Trp | W |
| Tyrosine | Tyr | Y |

Transitions vs Transversion Diagram

Transition

Transversion

Cytosine

Thymine

Guanine

Adenine
